# Genome-wide methylation biomarkers and biological aging in patients with bipolar disorder characterized for lithium response

**DOI:** 10.1192/j.eurpsy.2025.10112

**Published:** 2025-10-01

**Authors:** Claudia Pisanu, Alessandra Minelli, Donatella Congiu, Anna Meloni, Pasquale Paribello, Lisa Buson, Caterina Chilllotti, Marco Bortolomasi, Raffaella Ardau, Massimo Gennarelli, Giovanni Severino, Federica Pinna, Marco Pinna, Martina Contu, Maria Del Zompo, Bernardo Carpiniello, Mirko Manchia, Alessio Squassina

**Affiliations:** 1 Section of Neuroscience and Clinical Pharmacology, Department of Biomedical Sciences, University of Cagliari, Cagliari, Italy; 2 Department of Molecular and Translational Medicine, University of Brescia, Brescia, Italy; 3 Genetics Unit, IRCCS Istituto Centro San Giovanni di Dio Fatebenefratelli, Brescia, Italy; 4 Section of Psychiatry, Department of Medical Sciences and Public Health, University of Cagliari, Cagliari, Italy; 5 Unit of Clinical Pharmacology, University Hospital Agency of Cagliari, Cagliari, Italy; 6 Psychiatric Hospital “Villa Santa Chiara”, Verona, Italy; 7 Unit of Clinical Psychiatry, University Hospital Agency of Cagliari, Cagliari, Italy; 8 Department of Pharmacology, Dalhousie University, Halifax, NS, Canada

**Keywords:** biomarkers, epigenetic age, mood disorders, precision psychiatry, transcriptional age

## Abstract

**Background:**

Epigenetic mechanisms might play a role in modulating susceptibility to bipolar disorder (BD) and response to lithium, the mainstay treatment for BD. Additionally, individuals with BD experience accelerated biological aging.

**Methods:**

We compared blood DNA methylation profiles measured with EPIC v.2.0 arrays between patients with BD (33 lithium responders and 31 nonresponders) and nonpsychiatric controls (*n* = 32), as well as based on long-term lithium response. In addition, we compared cellular aging between these groups using epigenetic age, pace of aging, and, for the first time, transcriptional age acceleration based on bulk RNA sequencing in 93 patients and 56 controls.

**Results:**

We identified 191 differentially methylated positions (DMPs) and 8 differentially methylated regions between patients with BD and controls, located in genes enriched for “Postsynaptic Density” (odds ratio = 6.81, *p* = 0.001). No DMP was significantly associated with lithium response after multiple testing correction. Patients showed a significantly higher biological age acceleration than controls based on two epigenetic clocks (GrimAge, Mann–Whitney *U* = 551, *p* = 0.0009; GrimAge2: *U* = 477, *p* = 9.0E-05) and pace of aging (DunedinPACE, *t* = 3.01, *p* = 0.003), but not on transcriptional age. While we observed no significant difference in epigenetic aging based on lithium response, lithium responders showed lower epigenetic acceleration using all clocks, with a trend observed using the PhenoAge clock (*t* = 1.97, *p* = 0.053).

**Conclusions:**

Our findings point to methylation patterns characterizing BD and support the hypothesis of accelerated cellular aging in BD.

## Introduction

Bipolar disorder (BD) is a recurrent psychiatric disorder with a prevalence of more than 1% of the global population [1]. BD represents one of the leading causes of disability among young people, often characterized by functional impairment and reduced quality of life [1]. In addition, BD is associated with premature mortality compared to the general population, mainly due to increased prevalence of comorbid disorders associated with aging, such as cardiovascular and metabolic conditions [2]. Lithium is the gold standard for the long-term management of BD, due to its efficacy in the reduction of recurrences, as well as in its specific antisuicidal properties [3]. However, only one-third of patients show an excellent response to lithium, with the other two-thirds showing partial or no response [3]. Despite the extensive agreement among the international guidelines, which indicate lithium as first-line treatment for maintenance in BD [4], its use has been declining over the last decade [5]. The factors involved in the observed reduction in lithium prescription are complex to identify, but it is reasonable to assume that the narrow therapeutic window and its propensity to induce severe side effects have contributed significantly. On the other hand, because lithium-responsive BD patients return to well-being with levels of functioning and quality of life indistinguishable from those of healthy individuals, the search for biological markers of lithium response has been intense in the last few decades. Indeed, the identification of reliable biomarkers would allow for earlier identification of patients who would benefit most from lithium therapy, thereby reducing the risk of side effects and shortening the time needed for effective disease management.

Despite the growing body of molecular research on lithium response, and the findings supporting the heritability of lithium response [6], to date, clinical features seem to constitute the most robust factors to distinguish responders from nonresponders. Indeed, excellent responders represent a subtype of BD with distinct clinical features, such as familial loading for BD, episodic course of illness, and absence of rapid cycling [7]. Nevertheless, clinical characteristics have inherent limitations as orthogonal predictors of lithium response since some of these are time-dependent and volatile (clinical course) and might not be identifiable early in the course of the illness, or need reliable information from collaterals (family history), which need to be established accurately with detailed in-person assessments. In addition, clinical markers alone might not reach an adequate discriminative power to classify lithium responders versus nonresponders [8]. Thus, biological markers that are stable and less prone to variation over time are extremely relevant in increasing the accuracy of predictive models of lithium response, especially if integrated with clinical data.

A large body of research has tried to elucidate the genetic architecture of lithium response. However, this research has been hampered by the multiple pharmacodynamic effects of lithium that make it hard to understand which molecular mechanism underlies its clinical efficacy [3]. Both BD and lithium response are multifactorial phenotypes resulting from an interaction between genetic predisposition and environmental factors. Accordingly, genetic variants can only explain a small proportion of the interindividual variability in these phenotypes. Epigenetic mechanisms dynamically modulate gene expression in response to environmental stimuli and, therefore, represent promising candidates to account for at least part of this missing heritability [9]. In particular, DNA methylation, that is, the transfer of a methyl group to the C5 position of a cytosine to form 5-methylcytosine, is one of the most studied epigenetic mechanisms, being the most stable form of epigenetic alteration [10].

Differences in global methylation [11], in the methylation status of candidate genes [12], or in genome-wide DNA methylation patterns [13] have been previously implicated in BD and in lithium response [14]. Marie-Claire et al. used a genome-wide approach (SeqCapEpi) and identified seven differentially methylated regions (DMRs) discriminating responders and nonresponders [15], three of which were subsequently validated with methylation-specific high-resolution melting in an extended sample [16]. A more recent study including 26 excellent responders and 27 nonresponders to lithium identified 130 differentially methylated positions (DMPs) and 16 DMRs between the two groups, with some of the identified genes previously implicated in lithium response [17].

Whole-genome methylation also enables the estimation of epigenetic age through epigenetic clocks. This is relevant since a large body of evidence suggests BD to be characterized by accelerated aging, a condition in which biological aging occurs at a faster rate than chronological aging [18]. Indeed, BD is characterized by decreased life expectancy (up to 10–20 years compared to the general population) and patients may show alterations of biological markers of cellular aging, such as shorter telomere length, altered mitochondrial DNA copy number, and increased brain age [18]. Some studies reported longer telomere length in patients treated with lithium compared with patients not treated with this drug or controls, or a positive correlation between telomere length and years of lithium treatment, supporting the hypothesis of a protective effect of lithium against accelerated aging [19-24]. Nonetheless, previous studies estimating epigenetic age in patients with BD, or the potential effect of pharmacological treatment, provided contrasting results. Two studies found no evidence of differences in epigenetic age acceleration in patients with BD compared with controls in lymphoblastoid cell lines [25] or in whole blood from lithium responders compared with nonresponders [17]. Conversely, another study showed epigenetic age deceleration in BD patients compared with controls, as well as in BD patients treated with combinations of mood stabilizers (including lithium, valproate, and carbamazepine) compared with patients taking no medication or treated in monotherapy [26]. Biological age can also be estimated through transcriptomics data, with this measure suggested to offer complementary information compared to epigenetic age [27]. However, to date, no study has explored transcriptional age in patients with BD.

In this study, we aimed to identify genome-wide methylation signatures differentiating (1) patients with BD and controls; and (2) patients with BD characterized as responders and nonresponders to long-term lithium treatment using a validated scale. In addition, we estimated epigenetic and transcriptional age to compare these groups in terms of biological age acceleration.

## Methods

### Sample

The sample for whole-genome methylation analysis included 64 euthymic patients with a diagnosis of BD type I or BD type II according to the criteria of the Diagnostic and Statistical Manual of Mental Disorders, 5th edition, and 32 nonpsychiatric controls with no personal or familial history of psychiatric disorders. Exclusion criteria for patients comprised comorbidity with severe organic disorders or with substance use disorders. Participants were recruited by trained clinical psychopharmacologists and psychiatrists at the Unit of Clinical Pharmacology and the Unit of Clinical Psychiatry of the University of Cagliari and the University Hospital Agency of Cagliari. Patients fulfilled the criteria for the evaluation of response to lithium according to the Retrospective Criteria of Long-Term Treatment Response in Research Subjects with Bipolar Disorder scale (Alda scale) [28]. Briefly, the scale quantifies the degree of improvement under treatment with a mood stabilizer with a total score (TS) from 0 to 10, adjusting for potential confounders. Patients with a TS ≥ 7 were defined as responders, as in previous studies [28, 29]. Thirty-three patients were responders, while 31 were nonresponders. Demographic and clinical variables were collected through direct interviews and assessment of available medical records and included age at recruitment, sex (defined as biological sex at birth), body mass index (BMI), age at onset, number of manic and depressive episodes lifetime, years of lithium treatment, and lithium treatment at blood draw. Demographic and clinical characteristics of the sample are shown in Table 1. Each participant provided a fasting peripheral blood sample drawn in the morning for DNA extraction. In an extended sample including 93 patients with BD and 56 nonpsychiatric controls (46 patients and 27 controls also included in the methylation analysis), a peripheral blood sample collected in PAXGene Blood RNA Tubes (Qiagen) was available for RNA extraction and bulk RNA sequencing. Demographic and clinical characteristics of this sample are shown in Supplementary Table 1. Participants were recruited using the same diagnostic criteria described for the methylation analysis at the same clinics previously described, as well as the Psychiatric Hospital “Villa Santa Chiara,” Verona. The research protocol followed the principles of the Declaration of Helsinki and was approved by the Ethics Committee of the University of Cagliari (Protocol numbers PG/2022/327 and 348/FC/2013), and the Ethics Committee of the province of Verona N: 4997/09.11.01). All participants signed informed written consent after a detailed description of the study procedures.

**Table 1.** Demographic and clinical characteristics of the sample for whole-genome methylation analysis

Variable	Patients with BD (*n* = 64)	Controls (*n* = 32)	Statistics	p
Age, mean years (SD)	54.13 (13.58)	53.38 (7.40)	*U* = 883	0.27
Sex (F/M)	41/23	18/14	*χ* ^2^ = 0.55	0.51
BMI, mean (SD)	26.65 (4.33)	23.88 (4.14)	*t* = 2.90	0.005

	Lithium responders(*n* = 33)	Lithium non-responders(*n* = 31)	Statistics	*p*
Age, mean years (SD)	55.58 (13.62)	52.58 (13.58)	*U* = 437	0.32
Sex (F/M)	22/11	19/12	*χ* ^2^ = 0.20	0.80
BMI, mean (SD)	26.30 (4.05)	26.94 (4.60)	*t* = −0.96	0.34
BD subtype (I/II/U)	19/14/0	20/8/3	*χ* ^2^ = 1.26	0.30
Age at onset, mean years (SD)	28.21 (9.49)	28.39 (8.73)	*t* = 0.08	0.94
Number of depressive episodes, mean (SD)	7.32 (7.13)	5.58 (6.29)	*t* = −1.02	0.31
Number of manic episodes, mean (SD)	6.13 (7.84)	4.29 (3.83)	*t* = −1.17	0.25
Lithium treatment at blood draw (Y/N)	33/0	28/3	*χ* ^2^ = 3.35	0.11
Lithium treatment, mean years (SD)	16.79 (9.89)	15.92 (13.09)	*t* = 0.67	0.51
Alda scale TS, mean (SD)	8.03 (1.05)	2.06 (2.19)	*U* = 0.00	<0.001

Abbreviations: BMI, body mass index; N, no; SD, standard deviation; TS, total score; U, unknown; Y, yes.

*Note:* Some variables were available for a subset of patients: BMI (57 patients, of which 26 responders and 31 nonresponders, and 31 controls); BD subtype (33 responders and 28 nonresponders); number of depressive or manic episodes (31 responders and 31 nonresponders); years of lithium treatment (29 responders and 21 nonresponders).

### Methylation assessment and quality control

Genomic DNA was extracted from whole blood using the salting-out method [30]. A total of 500 ng of genomic DNA was bisulfite-converted using the EZ DNA Methylation-Lightning Kit (Zymo Research). Genome-wide DNA methylation was measured with the Infinium Methylation EPIC v2.0 arrays (Illumina, San Diego, CA, USA) using an iScan system (Illumina) at the Centre for Research University Services (CeSAR) of the University of Cagliari. Preprocessing of IDAT files was conducted using the minfi [31] and the ChAMP packages [32] in R. Quality control included removal of probes with detection *p*-value <10e-16, with a bead count < 3 in at least 5% of samples, non-CpG probes, single-nucleotide polymorphisms-related probes, and probes located on X,Y chromosomes. Replicate probes were averaged with the DMRcate R package [33]. After quality control, analyses included 781,602 probes and 96 participants. Visual inspection of multidimensional scaling plots and of type-I and type-II density plots was conducted before and after Beta-Mixture Quantile normalization. The singular value decomposition (SVD) method was used to assess the association between the main sources of variation and the top principal components (PCs). Batch effect correction for the slide was conducted with the ComBat normalization method in ChAMP. Smoking was estimated based on M values of the cg05575921 probe, while cell type proportions were estimated using the Houseman algorithm [34].

### Analysis of differentially methylated probes and functional enrichment

We assessed DMPs between patients with BD and controls using general linear models implemented in limma [35] adjusting for sex, age, estimated smoking, the first five cell type proportions PCs, and the top five control probes PCs. Models assessing DMPs between lithium responders and nonresponders were adjusted for sex, age, estimated smoking, the first five cell type proportions PCs, and lithium treatment at recruitment. Covariates were selected due to their suggested effect on methylation profiles in previous studies or their association with methylation values on the first PCs in the SVD plot. Upstream regulators of significant DMPs were identified with Ingenuity Pathway Analysis (IPA) [36]. The blood–brain correlation of significant CpGs was tested with the BECon tool [37]. DMRs between groups were assessed with DMRcate [33]. *P*-values were computed based on Stouffer’s method and adjusted for multiple testing based on the Benjamini–Hochberg false discovery rate (FDR) procedure. [33]. Functional enrichment for Reactome pathways and Gene Ontology (GO) terms of genes in which DMPs and DMRs were located was conducted with enrichR [38] with default settings. For all analyses, results were adjusted for multiple testing based on FDR, and an adjusted *p* < 0.05 was considered to be significant.

### Analysis of epigenetic age acceleration

Five epigenetic clocks (Horvath1, Hannum, PhenoAge, GrimAge, and GrimAge2) and their respective epigenetic age acceleration values (i.e., the residual from regressing epigenetic age on chronological age) were computed with the DNA Methylation Age Calculator web portal [39, 40]. A positive epigenetic age acceleration indicates a higher epigenetic age compared to chronological age. Normality of distribution was tested with the Shapiro–Wilk test, and the correlation between epigenetic clocks or epigenetic age acceleration clinical variables was computed with Pearson’s or Spearman’s correlation test, based on the distribution of the tested variables, or with a zero-inflated Poisson model in the case of the Alda scale TS. Epigenetic age acceleration was used for all subsequent analyses. Two outliers for GrimAge and GrimAge2 epigenetic acceleration identified with Grubb’s test were removed. Unadjusted differences in epigenetic age acceleration between patients with BD and controls or lithium responders and nonresponders were tested with Student’s *t*-test or Mann–Whitney *U* test, based on the distribution. Since epigenetic acceleration based on the GrimAge and the GrimAge2 clocks showed a significant difference between patients and controls, its association with covariates was tested with Mann–Whitney *U* test (sex, BD subtype, and treatment with lithium at blood draw) or Spearman’s correlation test (quantitative variables). Analyses adjusted for variables showing a significant association with epigenetic age acceleration (sex) or potential confounders (chronological age and BMI) were conducted using Quade nonparametric analysis of covariance. Finally, we computed DunedinPACE, which is a blood DNA methylation biomarker of the pace of biological aging. Based on methylation levels of 173 CpG sites, DunedinPACE quantifies the pace of aging of an individual (i.e., the number of biological years aged in comparison to one chronological year of aging) [41]. Values >1 represent a faster pace of aging. We tested the unadjusted association between DunedinPACE and categorical or quantitative variables using Student’s *t*-test or Pearson’s correlation test, respectively. In addition, we tested the association between DunedinPACE and BD diagnosis using a binary logistic regression model with BD diagnosis as the outcome, and sex, chronological age, and BMI as predictors.

### Analysis of transcriptional age acceleration

Library preparation and bulk RNA sequencing were performed at CeSAR (University of Cagliari). Total RNA was extracted from 2.5 mL of blood with the PAXGene Blood miRNA Kit (Qiagen). RNA quality was evaluated using an RNA ScreenTape system (Agilent), and all samples had an RNA integrity number value ≥7. RNA samples were processed using the Illumina Stranded Total RNA Prep with Ribo-Zero Plus kit, and paired-end sequencing (2 × 150 bp) was performed on a NextSeq 2000 platform (Illumina). After quality control performed with FASTQC and MULTIQC, raw data were processed with the rnaseq nf-core pipeline [42]. Alignment with the reference genome (GRCh38) was performed with STAR [43], while gene expression levels were estimated with RSEM [44]. Normalized counts were used as input to compute transcriptional age with the RNAAgeCalc tissue-specific method [27]. This method was developed through the identification of age-related genes in the GTEx database and allows for computing transcriptional age acceleration (computed as the residuals from regressing transcriptional age on chronological age) in a tissue-specific way. A positive transcriptional age acceleration indicates higher transcriptional age compared to chronological age. The presence of outliers was checked with Grubb’s test, and no outliers were identified. The correlation between transcriptional age and chronological age was tested with Pearson’s correlation test or with a zero-inflated Poisson model in the case of the Alda scale TS. Unadjusted differences in transcriptional age acceleration between patients with BD and controls, or between lithium responders and nonresponders, were tested with Student’s *t*-test. The association between transcriptional age acceleration and covariates was tested with Student’s *t*-test or Pearson’s correlation test.

## Results

### Association between whole-blood methylation profiles and BD or lithium response

**Figure 1.**
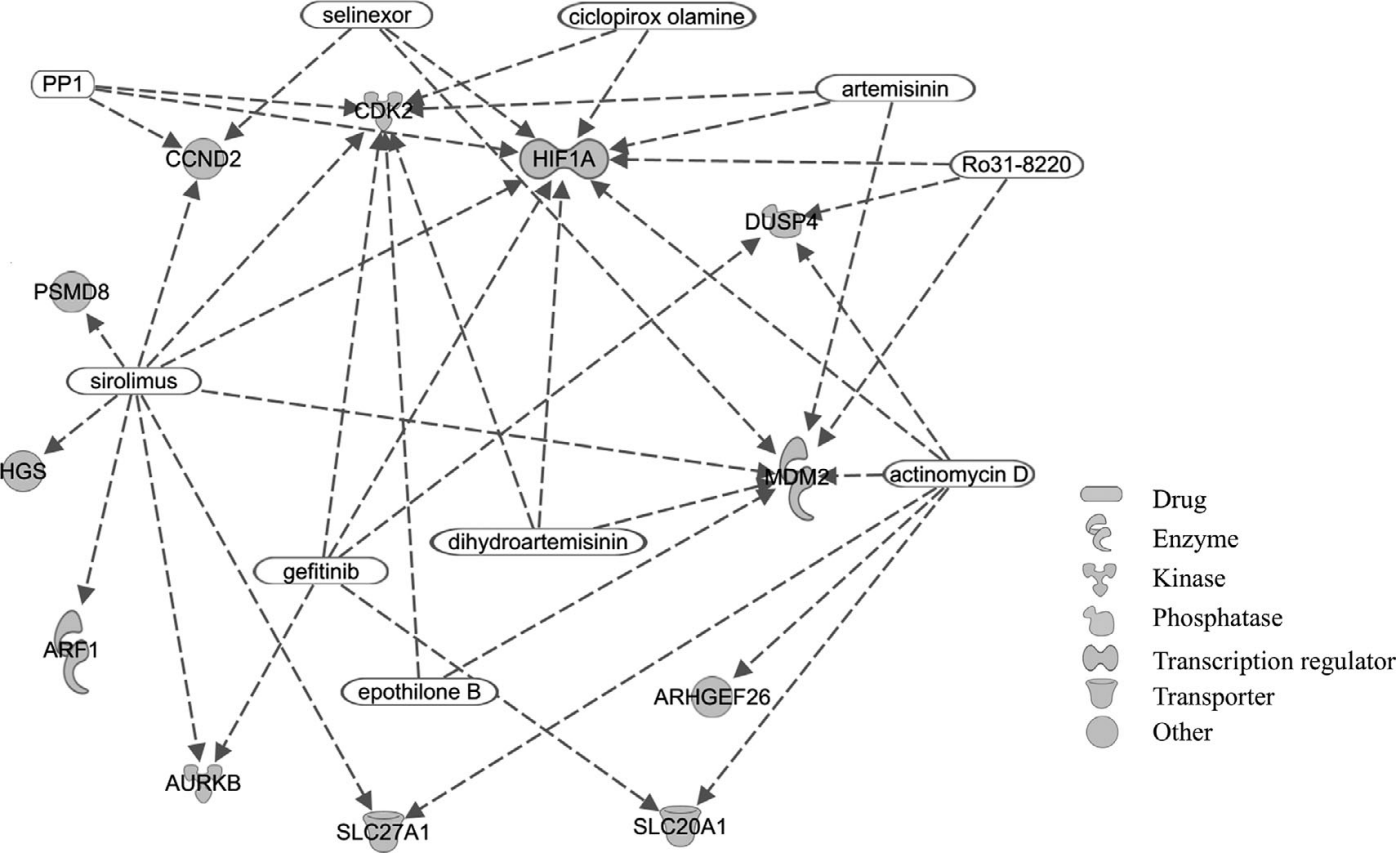
Network of drugs acting as significant upstream regulators of genes associated with bipolar disorder.

We identified 191 DMPs (Supplementary Table 2) and 8 DMRs (Table 2) between patients with BD and controls. Results for the top 30 DMPs are reported in Table 3, while the complete list of DMPs is shown in Supplementary Table 2. A total of 97 and 94 probes were hyper- and hypomethylated in patients with BD compared with controls, respectively. Eighty-five probes were located in genes, while the remaining probes were in intergenic regions. Genes in which DMPs and DMRs were located were enriched for three pathways related to cellular cycle as well as the “Serine/Threonine Protein Kinase Complex” cellular GO term (odds ratio [OR] = 12.4, adjusted *p* = 0.04), the “Postsynaptic Density” (OR = 6.8, adjusted *p* = 0.01), and the “Regulation of Postsynaptic Neurotransmitter Receptor Endocytosis” (OR = 15.7, adjusted *p* = 0.01) SynGO terms (Table 4). We identified 42 upstream regulators of genes in which significant DMPs were located (Supplementary Table 3). A network of significant upstream regulators classified as biological or chemical drugs and their regulated genes is shown in Figure 1. Using the BECon tool, we found that most of the CpGs associated with BD exhibited no or weak blood–brain correlation (percentile of mean positive blood–brain correlation <50%) (Supplementary Table 2). However, seven DMPs were in the 50–75% percentile of mean positive blood–brain correlation (of which four located in the NRM, MDM2, SFTA3, and CRYZL1 genes, and three intergenic), three in the 75–90% percentile (of which two located in the NFU1 and PDE4A genes, and one intergenic) and two in the 90% percentile (located in the RPS6KC1 and NOTCH4 genes). These results suggest that a subset of the significant CpGs might also be differentially methylated in the brain. While we did not identify any significant DMP between lithium responders and nonresponders after multiple testing correction, suggestive probes (*p*-values <1E-05) are reported in Supplementary Table 4. In addition, several genes, including DMPs identified in the study by Zafrilla-Lopez et al. (19 hypermethylated and 7 hypomethylated in responders) [17] or a DMR identified in the study by Marie-Claire et al. (EIF2B5) [15], showed nominal significance in our results and are reported in Supplementary Table 5.

**Table 2.** DMRs between patients with BD and controls

ID	Chr	Start	End	N.cpgs	FDR	Meandiff	Overlapping genes
DMR1	16	56961901	56961944	3	0.009	0.06	CETP
DMR2	17	48603455	48605418	15	0.019	−0.09	HOXB-AS3, HOXB3, HOXB6
DMR3	10	34636101	34636712	3	0.023	−0.04	PARD3
DMR4	1	226549544	226549640	2	0.030	0.01	STUM
DMR5	12	54369427	54369649	3	0.035	0.07	AC079313.2, AC079313.1, ZNF385A
DMR6	2	27306672	27306920	2	0.039	0.02	TRIM54
DMR7	3	308820	309320	6	0.041	−0.06	CHL1, RPS8P6
DMR8	7	74656163	74656567	2	0.043	−0.01	GTF2I

Abbreviations: Chr, chromosome; DMR, differentially methylated region; FDR, false discovery rate; N.cpgs, number of cpgs.

**Table 3.** Top 30 DMPs between patients with BD and controls

Probe	Chr	BP	logFC	t	p	FDR	Gene	Feature	Cgi
cg13259408	10	34636101	−0.44	−7.30	1.55E–10	0.0001			OpenSea
cg23550779	12	29372422	−0.74	−6.98	6.45E–10	0.0003			OpenSea
cg04948941	14	61695160	0.26	6.31	1.30E–08	0.0032	HIF1A	TSS1500	Island
cg08836736	11	30424053	−0.37	−6.25	1.66E–08	0.0032			OpenSea
cg01636258	4	80615269	−0.49	−6.17	2.38E–08	0.0035			OpenSea
cg26797661	1	151166040	0.26	6.14	2.69E–08	0.0035	SCNM1	TSS200	OpenSea
cg24395955	19	32362571	−1.29	−6.07	3.62E–08	0.0040			OpenSea
cg10120645	8	124474394	0.35	5.91	7.12E–08	0.0069	RNF139-DT	Exon	Island
cg20335273	13	51623731	0.60	5.89	7.90E–08	0.0069			OpenSea
cg11265407	7	80541942	−0.41	−5.86	9.02E–08	0.0071			OpenSea
cg01173986	1	62596297	−0.59	−5.76	1.39E–07	0.0093	ANGPTL3	TSS1500	OpenSea
cg02771392	7	64902704	−0.40	−5.75	1.43E–07	0.0093	ZNF273	TSS1500	OpenSea
cg25102879	10	95168442	−0.47	−5.71	1.69E–07	0.0096			OpenSea
cg00244474	1	12268366	−0.33	−5.69	1.82E–07	0.0096			OpenSea
cg24402392	11	123198430	−0.41	−5.69	1.85E–07	0.0096			Shelf
cg13236968	8	61454112	0.57	5.67	1.96E–07	0.0096			OpenSea
cg25744017	15	52527127	−0.26	−5.61	2.62E–07	0.0121			Shore
cg23989618	19	4992235	−0.85	−5.59	2.80E–07	0.0122			OpenSea
cg16667784	11	78257237	−0.34	−5.51	3.94E–07	0.0143			OpenSea
cg23668004	7	4745272	0.29	5.51	3.95E–07	0.0143			Island
cg18608814	8	51617923	−0.49	−5.51	3.96E–07	0.0143			OpenSea
cg07914296	2	188296928	−0.51	−5.50	4.02E–07	0.0143	MIR561	TSS1500	Shelf
cg25568561	14	101809750	0.48	5.47	4.60E–07	0.0146	PPP2R5C	TSS200	OpenSea
cg08105772	18	74267042	−0.38	−5.46	4.84E–07	0.0146			OpenSea
cg14519123	15	66252627	0.25	5.46	4.86E–07	0.0146			Island
cg11921411	3	61965316	−0.76	−5.45	4.91E–07	0.0146			OpenSea
cg25216443	2	177771466	−0.84	−5.43	5.40E–07	0.0146			OpenSea
cg16705351	1	226549544	0.33	5.43	5.47E–07	0.0146			Island
cg02996269	4	105473535	0.19	5.43	5.49E–07	0.0146			Island
cg24217756	3	180989722	0.47	5.42	5.61E–07	0.0146	DNAJC19	5UTR	Island

**Table 4.** Functional enrichment for Reactome pathways and GO terms for genes located in DMPs and DMRs associated with BD

Term	Description	P	FDR	Odds ratio	Genes
**Reactome pathways**					
R-HSA–69563	p53-dependent G1 DNA damage response	0.0001	0.03	17.24	PSMD8, CDK2, MDM2, ZNF385A
R-HSA–69580	p53-dependent G1 S DNA damage checkpoint	0.0001	0.03	17.24	PSMD8, CDK2, MDM2, ZNF385A
R-HSA–69615	G1 S DNA damage checkpoints	0.0002	0.03	16.56	PSMD8, CDK2, MDM2, ZNF385A
**GO cellular component**					
GO:1902554	Serine/threonine protein kinase complex	0.0004	0.04	12.41	CCNJ, CCND2, PARD3, CDK2
**SynGO 2024**					
GO:0014069	Postsynaptic density	0.0012	0.01	6.81	ARF1, RPS6KC1, MDM2, PIP5K1C, PHB2
GO:0099149	Regulation of postsynaptic Neurotransmitter Receptor Endocytosis	0.0012	0.01	15.68	MDM2, PIP5K1C, PACSIN1

### Differences in epigenetic age acceleration

**Figure 2.**
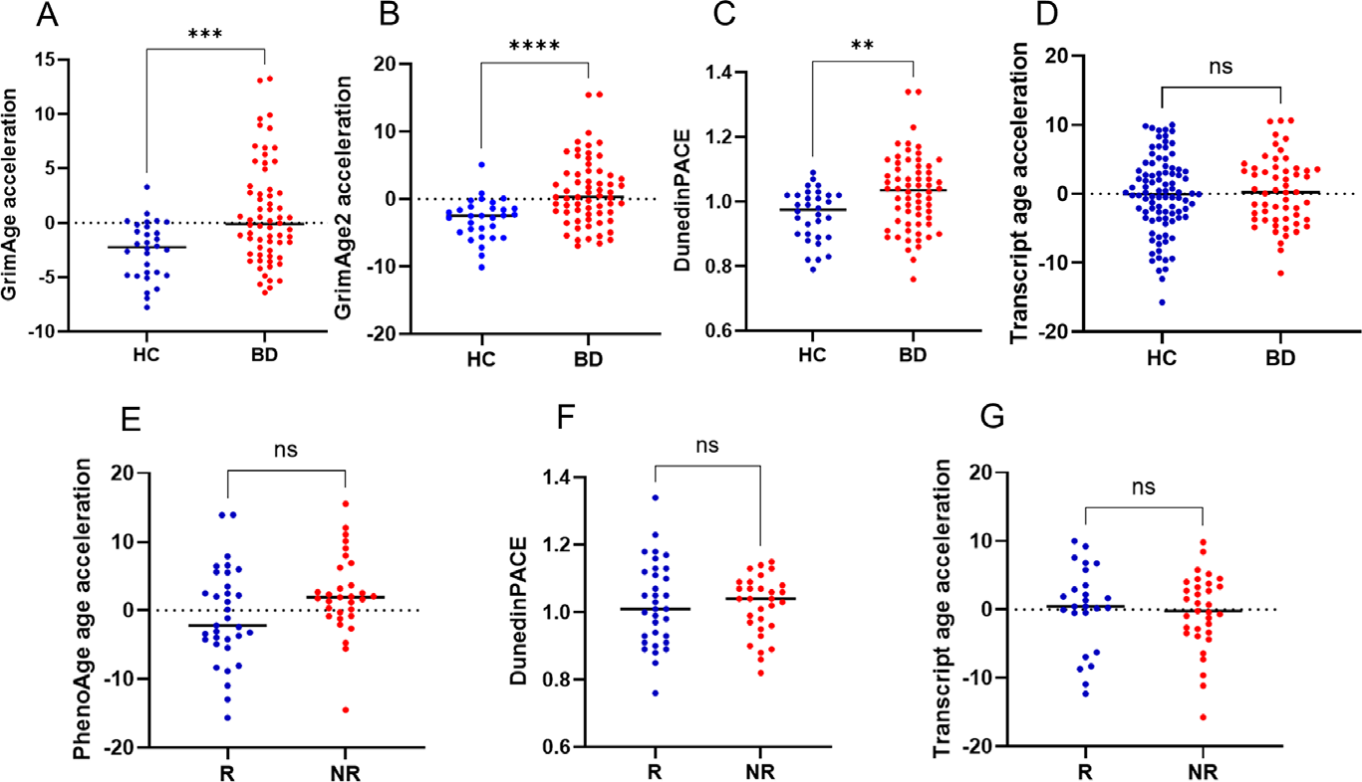
Differences in epigenetic and transcriptional age acceleration between BD and controls or between lithium responders and nonresponders. The upper part of the figure shows differences between patients with BD and controls in (A) epigenetic age acceleration using the GrimAge or (B) the GrimAge2 epigenetic clock, (C) DunedinPACE, and (D) transcriptional age acceleration; the lower part of the figure shows differences between lithium responders and nonresponders in (E) epigenetic age acceleration using the phenoAge epigenetic clock, (F) DunedinPACE, and (G) transcriptional age acceleration. BD, bipolar disorder; HC, healthy controls; NR, nonresponders; ns, not significant; R, responders.

All epigenetic clocks were strongly and positively correlated with chronological age (Supplementary Table 6). Patients with BD showed a significantly higher epigenetic age acceleration than controls, only based on the GrimAge (*U* = 551, *p* = 0.0009) and GrimAge2 (*U* = 477, *p* = 9.0E-05) epigenetic clocks (Figure 2), although the same direction of effect was observed for all clocks (Supplementary Table 7). GrimAge and GrimAge2 epigenetic age acceleration were significantly lower in women than in men, while no association with other clinical variables was detected (Supplementary Table 9). When conducting Quade analysis of covariance adjusted for sex, chronological age, and BMI, the difference between patients and controls was still significant with either the GrimAge (F_1,84_ = 14.30, *p* = 0.0003) or the GrimAge2 (F_1,84_ = 16.30, *p* = 0.0001) epigenetic clocks. Although we did not detect a significant association between epigenetic age acceleration and lithium response, lithium responders showed lower epigenetic age acceleration than nonresponders using all clocks (Supplementary Table 8), and a trend for significance was observed using the PhenoAge epigenetic clock (*t* = 1.97, *p* = 0.053).

In addition, patients with BD showed a faster pace of aging based on DunedinPACE compared with controls (BD: 1.03, controls: 0.96, *t* = 3.01, *p* = 0.003, Figure 2). The pace of aging was significantly positively correlated with BMI (Pearson’s correlation coefficient = 0.30, *p* = 0.005) and not significantly associated with other variables (Supplementary Table 9). The association between BD and DunedinPACE was significant also in a binary logistic regression model (R2 di Nagelkerke = 0.26, *p* of the model 0.006, DunedinPACE *p* = 0.029) adjusted for sex (0.023), chronological age (0.22), and BMI (*p* = 0.006). We observed no significant difference in the pace of aging measured with DunedinPACE based on lithium response (*t* = −0.24, *p* = 0.81).

### Differences in transcriptional age acceleration

Transcriptional age was positively correlated with chronological age (Pearson’s correlation coefficient = 0.60, *p* = 0.001) and with epigenetic age using all clocks, while only a trend for positive correlation was observed for the pace of aging estimated with DunedinPACE (Supplementary Table 10). However, we did not find a significant difference based on BD diagnosis (*t* = 0.61, *p* = 0.54) or lithium response (*t* = 0.45, *p* = 0.66). Transcriptional age acceleration was not associated with other clinical variables (Supplementary Table 11).

## Discussion

This study investigated genome-wide peripheral blood DNA methylation patterns and epigenetic and transcriptional age acceleration in individuals with BD compared to nonpsychiatric controls, and explored differences based on lithium response within the BD group. Our findings showed significant alterations in DNA methylation in BD patients compared to controls, providing novel insights into the molecular underpinnings of this complex mental disorder. Moreover, they further support the evidence that BD might be associated with accelerated aging.

The set of DMPs and DMRs associated with BD was enriched for different pathways and GO terms, among which two were related to postsynaptic density, an electron-dense structure containing a high concentration of scaffolding, adaptor, and signaling proteins, adjacent to the postsynaptic membrane of excitatory synapses [45, 46]. This macromolecular complex is involved in several functions, including dopamine and glutamate-dependent synaptic plasticity processes at excitatory synapses [47]. Genes involved in postsynaptic density have been implicated in the pathophysiology of BD by a study integrating brain transcriptome data from postmortem dorsolateral prefrontal cortex and genome-wide association (GWA) signals [48]. Using weighted gene co-expression network analysis, the authors found that modules including genes differentially expressed in BD and with signals in prior GWA of BD were enriched for genes involved in postsynaptic density, strongly supporting the hypothesis that dysregulation of this structure might be involved in the pathogenesis of BD.

Using IPA, we identified a network of upstream regulators of genes including DMPs significantly associated with BD (Supplementary Table 3). Chemical compounds in the network included artemisinin and its derivative dihydroartemisinin (Figure 1 and Supplementary Table 3). Artemisinin is a natural antimalarial compound obtained from the *Artemisia annua* plant. Artemisin and dihydroartemisinin are currently being investigated in autoimmune disorders due to their potential immunomodulatory effects [49, 50]. In addition, artemisinin has been found to be able to suppress neuroinflammation and promote synaptic plasticity in rats subjected to single prolonged stress [51], and dihydroartemisinin to exert antidepressant effects in chronic unpredictable mild stress-induced mice [52]. These compounds were found to be upstream regulators of three genes differentially methylated in our data, two of which were hypermethylated (CDK2 and HIF1A) and one hypomethylated (MDM2) in patients with BD compared with controls (Supplementary Tables 2 and 3). While the potential repurposing properties of artemisinin in psychiatric disorders have just started to be explored in preclinical models, our results support its potential role as a regulator of genes differentially methylated in patients with BD compared with controls.

While we did not identify DMPs significantly associated with lithium response after multiple testing correction, several genes including DMPs identified by Zafrilla-Lopez et al. (hypermethylated in responders: ABI2, ALK, B4GALNT3, CACNA2D2, CD19, CNIH3, HP1BP3, KCNQ5, NEUROD4, NHSL1, PSME3, RIMBP2, SLC45A2, SYTL3, TENM2, TEP1, THSD7A, ZC3H4, and ZNHIT3; or hypomethylated in responders: ADAMTS12, PDE2A, RGS10, TMEM177, TMEM201, TRAF3IP2-AS1, and ZNF205) [17] or a DMR identified by Marie-Claire et al. (EIF2B5) [15], showed nominal significance in our results (Supplementary Table 5). While the lack of significance after multiple testing correction might be explained by several factors, including the limited sample size, clinical heterogeneity, as well as the methods used to measure methylation compared with previous studies, the nominal significance of a large number of targets with the same direction of effect supports the hypothesis of a role of epigenetic mechanisms in lithium response.

We observed higher epigenetic age acceleration and pace of aging, but not transcriptional age acceleration, in patients with BD compared with controls. Premature senescence has been largely supported in BD, based on studies suggesting a higher risk of developing dementia [53], premature death [54, 55], accelerated deterioration in executive functions and cognitive regulation [56], comorbid medical conditions typically associated with aging [57], and a faster rate of brain aging [58, 59]. In terms of molecular findings, several studies reported signatures of accelerated biological aging in BD, such as shorter telomere length and altered telomere dynamics [21], accelerated brain aging in imaging studies [60], increased inflammation and oxidative stress [61], altered DNA mitochondrial copy number [62, 63], genetically determined aging markers [64], as well as accelerated epigenetic age [65]. For the first time, we investigated increased transcriptional age acceleration in patients with BD compared with controls. While transcriptional age has only recently started to be explored in psychiatric disorders, due to the relative paucity of suitable methods and of transcriptome data, this measure has been suggested to offer complementary information to DNA methylation age in the analysis of mortality risk across different types of cancer [27]. This method has recently allowed for determining higher biological age in individuals with post-traumatic stress disorder (PTSD) in blood [66] as well as individuals with PTSD or major depressive disorder in post-mortem brain samples from the ventromedial prefrontal cortex, compared to controls [67]. To our knowledge, our study is the first to compute transcriptional age in BD, thus contributing valuable data that supports the exploration of biological aging in mental illness through alternative and complementary methods. While we observed no significant difference in epigenetic aging based on lithium response, in line with a previous study [17], lithium responders showed lower epigenetic acceleration using all clocks, with a trend more evident using the PhenoAge epigenetic clock (*t* = 1.97, *p* = 0.053). While this trend has to be interpreted cautiously, it is in line with the hypothesis that lithium might exert protective properties against accelerated cellular aging in patients with BD [68].

Strengths of our study include the use of a validated scale to characterize lithium response and the use of two complementary measures of biological aging. However, our results should be interpreted considering some limitations. Whole genome methylation analysis and bulk RNA sequencing were conducted on whole blood DNA and RNA. We found a significant enrichment for the “postsynaptic density” and “Regulation of postsynaptic neurotransmitter receptor endocytosis” SynGO terms, as well as a subset of DMPs in the highest percentiles of brain–blood correlation based on BECon, suggesting a potential brain relevance for at least some of the identified targets. However, while the access to brain DNA methylation and RNA expression remains difficult, studies on neural precursors or differentiated neurons from induced pluripotent stem cells might allow us to elucidate whether our results might be relevant for central mechanisms or rather be limited to the identification of peripheral biomarkers of diagnosis and clinical response. In addition, this study only included participants of European origin. Therefore, the extent to which results might be extended to different populations has not been explored. Finally, we did not validate targets identified by previous studies investigating genome-wide methylation in patients with BD characterized for lithium response. Although various factors may have contributed to the lack of replication (e.g., variability in experimental design or differences in clinical recruitment procedures across studies), one key factor could be the limited sample size after stratification by lithium response – even though this is consistent with previous studies [15, 17].

In conclusion, our results point to different peripheral methylation profiles associated with BD and support the hypothesis of accelerated aging in BD. Moreover, pathway analysis of identified targets suggested that some of the DMPs are targeted by natural compounds with immunomodulatory and antidepressant effects, thus potentially supporting drug repurposing strategies for a more effective management of BD.

## Supporting information

10.1192/j.eurpsy.2025.10112.sm001 Pisanu et al. supplementary material Pisanu et al. supplementary material

## Data Availability

De-identified data are available from the corresponding author upon reasonable request.

## Supplementary material

The supplementary material for this article can be found at http://doi.org/10.1192/j.eurpsy.2025.10112.
